# Mirincamycin, an old candidate for malaria combination treatment and prophylaxis in the 21st century: *in vitro* interaction profiles with potential partner drugs in continuous culture and field isolates

**DOI:** 10.1186/1475-2875-13-228

**Published:** 2014-06-10

**Authors:** Peter Starzengruber, Hans-Peter Fuehrer, Paul Swoboda, Deepa Ganesh, Rashidul Haque, Wasif A Khan, Wolfgang Graninger, Harald Noedl

**Affiliations:** 1 Institute of Specific Prophylaxis and Tropical Medicine, Medical University of Vienna, Vienna, Austria; 2 MARIB, Malaria Research Initiative Bandarban, Bandarban, Bangladesh; 3 Department of Hospital Hygiene and Infection Control, Vienna General Hospital, Medical University Vienna, Vienna, Austria; 4 Department of Pathobiology, Institute of Parasitology, University of Veterinary Medicine Vienna, Vienna, Austria; 5 Institute of Medical Chemistry, Department of Biochemistry and Genetics, Medical University of Vienna, Vienna, Austria; 6 International Centre for Diarrhoeal Disease Research, Bangladesh, Dhaka, Bangladesh; 7 Department of Medicine I, Division of Infectious Diseases and Tropical Medicine, Medical University of Vienna, Vienna, Austria

**Keywords:** Mirincamycin, *In vitro*, Malaria, Interaction profile, *Plasmodium falciparum*, Resistance, Tafenoquine, Chloroquine

## Abstract

**Background:**

Spreading resistance of *Plasmodium falciparum* to existing drugs calls for the search for novel anti-malarial drugs and combinations for the treatment of falciparum malaria.

**Methods:**

*In vitro* and *ex vivo* investigations were conducted with fresh *P. falciparum* field isolates and culture-adapted *P. falciparum* clones to evaluate the anti-malarial potential of mirincamycin, a lincosamide, alone and in combination with tafenoquine (TQ), dihydroartemisinin (DHA), and chloroquine (CQ). All samples were tested in a histidine-rich protein 2 (HRP2) drug susceptibility assay.

**Results:**

Interaction analysis showed additive to synergistic interaction profiles with these potential partner drugs, with an overall geometric mean fractional inhibitory concentration at 50% inhibition (FIC_50_) of 0.78, 0.80 and 0.80 for mirincamycin with TQ, DHA, and CQ, respectively. Antagonism was not found in any of the tested field isolates or clones. The strongest tendency toward synergy (i.e. the lowest FIC) was seen with a combination ratio of 1:0.27 to 1:7.2 (mean 1:2.7) for the combination with tafenoquine. The optimal combination ratios for DHA and CQ were 1:444.4 to 1:36,000 (mean 1:10,755.5) and 1:2.7 to 1:216 (mean 1:64.5), respectively. No evidence of an activity correlation (i.e. potential cross-resistance) with DHA, mefloquine, quinine or chloroquine was seen whereas a significant correlation with the activity of clindamycin and azithromycin was detected.

**Conclusions:**

Mirincamycin combinations may be promising candidates for further clinical investigations in the therapy and prophylaxis of multidrug-resistant falciparum malaria or in combination with 4 or 8-aminoquinolines for the treatment and relapse prevention of vivax malaria.

## Background

Artemisinin-based combination therapy (ACT) has been adopted as first-line treatment for *Plasmodium falciparum* malaria in virtually all malaria-endemic countries. In the view of spreading anti-malarial drug resistance and the emergence of the first cases of compromised susceptibility to artemisinins along the Thai-Cambodian border the development of novel compounds and combinations for the treatment of falciparum malaria has become an issue of utmost importance [1,2]. First studies conducted in 1969 and 1970 in rhesus monkeys infected with *Plasmodium cynomolgi*, a malaria parasite of monkeys similar to *Plasmodium vivax,* have shown that mirincamycin, a synthetically-produced lincosamide antibiotic similar to clindamycin, has substantial antiplasmodial activity for prophylaxis as well as for radical cure in animal models [3,4]. Moreover in the above-mentioned *P. cynomolgi* model mirincamycin was shown to be superior compared to clindamycin in both, prophylaxis and radical cure. In 1985 Schmidt *et al.* showed that concomitant administration of mirincamycin improved the hypnozoitocidal efficacy of primaquine in a *P. cynomolgi* model suggesting that the primaquine dose required for radical cure could be reduced by one-half to two-thirds by coadministration with mirincamycin [5]. In 2010, Held *et al.*[6] assessed the *in vitro* activity of mirincamycin in *P. falciparum* field isolates from Gabon and indicated that mirincamycin is more active than the comparator drugs clindamycin and doxycycline. In Gabon, the observed mean IC_50_ measure after six days of incubation was 10,000-fold lower than on day 3, suggesting a slow onset of action (delayed death phenomenon) as previously described for several other antibiotics [6-8]. A recent study by Khemawoot *et al.* found a more than 100-fold increased potency against field isolates of *P. falciparum* cultured *ex vivo* in primate plasma compared to *in vitro* investigations with the W2 clone [9]. A challenge for the radical cure of *P. vivax* remains the haemolytic toxicity of primaquine in G6PD-deficient individuals. Earlier studies suggest that mirincamycin may improve the efficacy of 8-aminoquinolines, thereby potentially allowing for lower doses and reducing toxicity when given in combination [5].

Therefore, mirincamycin has been investigated as a promising combination partner for prophylaxis and treatment of falciparum malaria.

## Methods

### Study area

The study was carried out at the MARIB (Malaria Research Initiative Bandarban) field site in Bandarban in southeastern Bangladesh and at the laboratories of the Medical University of Vienna, Austria.

### Ethics

Written informed consent was obtained from all study participants or their legal representatives and the study protocol was approved by the Ethical Review Committees of the ICDDR,B and the Medical University of Vienna.

### Sampling

Blood samples were taken from male and non-pregnant female patients aged 8 to 65 years, presenting with microscopically confirmed *P. falciparum* monoinfections and parasite densities between 1,000 and 100,000 asexual parasites per μl. Blood samples exceeding 1% parasitemia were diluted with uninfected red blood cells before testing. Pregnant or breastfeeding women and patients with anti-malarial drug therapy in the preceding 30 days were excluded from participation.

### Drug susceptibility testing

Mirincamycin (4’-trans-mirincamycin hydrochloride, molecular weight [MW] 475.47) was provided by Richard Westerman (Maldevco), tafenoquine (MW 463.49) by GlaxoSmithKline (GSK), clindamycin hydrochloride (MW 461.44), azithromycin (MW 748.98), dihydroartemisinin (MW 284.3), chloroquine diphosphate (MW 515.90), quinine sulphate (MW 782.96) and mefloquine hydrochloride (MW 414.77) were obtained from Sigma Aldrich. All drugs were dissolved in 70% ethanol to obtain a 1 mg/ml stock solution.

All samples were cultured for 72 hours to allow for a direct comparison among all test substances and combinations and growth inhibition assessed using the histidine-rich-protein 2 (HRP2) *in vitro* drug susceptibility assay. The culture and enzyme linked immunosorbent assay (ELISA) were performed as previous described [8,10,11].

To investigate interaction profiles and most effective ratio of concentrations of mirincamycin in combination with standard anti-malarials, fresh *P. falciparum* field isolates and culture-adapted *P. falciparum* clones K1 (chloroquine-resistant) and 3D7 (chloroquine-sensitive) were tested in checkerboard assays and data analysis was done as previously described [11-13]. Checkerboards assessing optimum concentrations of various drugs in combination were performed by diluting trans-mirincamycin (MIR 27.43 to 20,000 ng/ml) vertically and either chloroquine (CQ 3.43 to 2,500 ng/ml), tafenoquine (TQ 34.29 to 25,000 ng/ml) or dihydroartemisinin (DHA 0.02 – 15 ng/ml) horizontally in a three-fold serial dilution using a standard 8x8 well design on microtiter plates.

In addition single compounds were testes with fresh *P. falciparum* field isolates in the presence of three-fold serial dilutions of the anti-malarial drugs *trans*-mirincamycin (MIR; 137.2 to 100,000 ng/ml), clindamycin (CLM; 68.6 to 50,000 ng/ml), azithromycin (AZM; 68.6 to 50,000 ng/ml), dihydroartemisinin (DHA; 0.034 to 25.0 ng/ml), chloroquine (CHL; 3.4 to 2,500 ng/ml), quinine (QNN; 3.4 to 2,500 ng/ml) and mefloquine (MEF; 0.3 to 250 ng/ml) and subsequently growth inhibition was quantified in a HRP2 ELISA. In a subset of samples *trans*-mirincamycin and clindamycin were also tested after 24 h incubation using the WHO microtest [14].

### Data analysis

The 50 and 90% inhibitory concentrations (IC_50s_ and IC_90s_, respectively) were calculated from optical density readings by nonlinear regression analysis. ICs were used to calculate fractional inhibitory concentrations (FICs) as previously described [11,13]. Isobolograms were plotted to demonstrate synergism (FIC < 0.5) and/or antagonism (FIC > 2) for drug combinations. Activity correlations were calculated by nonparametric correlation analysis (Spearman).

## Results

**Figure 1:**
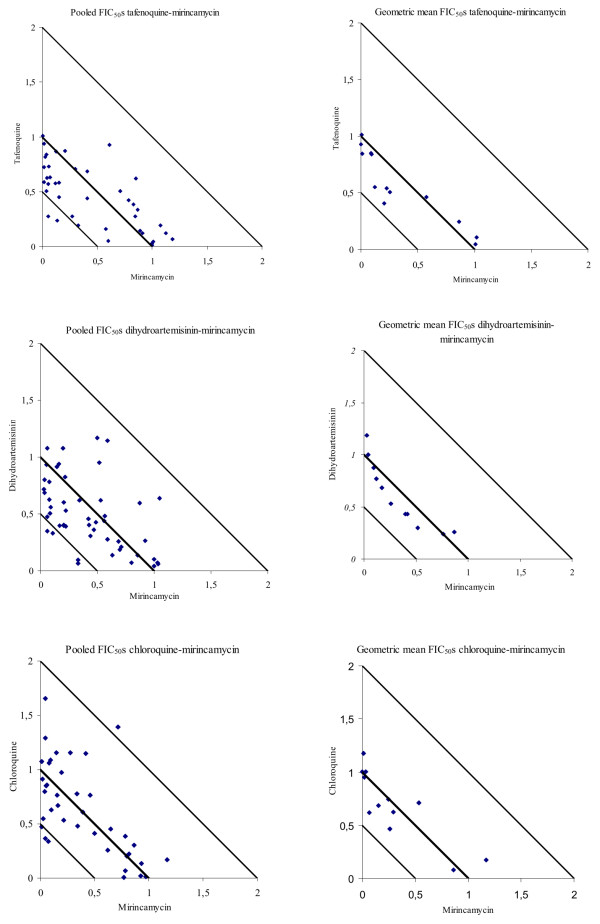
**Isobolograms of mirincamycin in combination with tafenoquine, dihydroartemisinin and chloroquine.** Isobolograms for the checkerboards assays of mirincamycin in combination with tafenoquine, dihydroartemisinin and chloroquine at various concentration ratios for all successfully tested isolates at FIC_50_ level. All individual data points (pooled FIC_50_s) at various concentration ratios are presented on the left hand side, isobolograms on the right side point out the geometric mean of the individual data points. The straight lines in each panel are representing the type of interaction. Data points below the 0.5-line show synergism (FIC < 0.5), around the 1.0-line are additive (FIC 0.5-2) and above the 2-line are antagonistic (FIC > 2).

Results of mirincamycin in combination with DHA, CQ and TQ are shown in Table 1 and Figure 1. All combinations with mirincamycin were additive with a slight trend to synergism, with an overall geometric mean fractional inhibitory concentration at 50% inhibition (FIC_50_) of 0.78, 0.80 and 0.80 for TQ, DHA, and CQ, respectively. Antagonism was not detected for any of the tested field isolates or clones. The highest level of synergism (i.e. the lowest FICs) was found for the combination of tafenoquine with mirincamycin at a ratio of 1:0.27 to 1:7.2 (mean 1:2.7). The optimal combination ratios were 1:444.4 to 1:36,000 (mean 1:10,755.5) and 1:2.7 to 1:216 (mean 1:64.5) for DHA and CQ, respectively.

**Table 1:** Results of mirincamycin in combination with DHA, CQ and TQ

	**IC**_**50 **_**(nM)**		**IC**_**90 **_**(nM)**		**ΣFIC**_**50**_^**a**^	**ΣFIC**_**90**_^**a**^
**Tafenoquine – mirincamycin (n)**	**TQ**	**MIR**	**TQ**	**MIR**		
Field isolates (n^b^ = 3)	2,962.85	2,357.12	9,027.36	15,653.18	0.56	0.50
3D7 (n^c^ = 3)	1,348.48	19,539.36	2728.49	36,936.11	0.86	1.09
K1 (n^c^ = 2)	1,076.23	24,736.61	5,271.96	39,284.24	0.88	1.03
All tested isolates (n = 8)	1,829.64	8,135.69	5,005.97	25,786.65	0.78	0.83
**DHA – mirincamycin** (n)	DHA	MIR	DHA	MIR		
Field isolates (n^b^ = 4)	3.23	1,067.34	13.0	20,101.72	0.81	0.69
3D7 (n^c^ = 3)	1.90	16,995.36	4.78	34,781.20	0.86	0.85
K1 (n^c^ = 2)	1.09	8,669.49	2.86	27,749.81	0.71	0.95
All tested isolates (n = 9)	2.12	4,276.94	6.65	25,925.12	0.80	0.83
**Chloroquine – mirincamycin** (n^b^)	CHL	MIR	CHL	MIR		
Field isolates (n^b^ = 2)	128.99	1,365.01	680.90	16,161.82	0.39	0.99
3D7 (n^c^ = 4)	28.27	23,288.21	63.87	38,311.62	1,06	1.04
K1 (n^c^ = 2)	651.93	18,869.50	1,174.69	36,535.66	0.95	0.99
All tested isolates (n = 8)	90.54	10,871.57	239.01	30,511.74	0.80	0.91

^a^Synergy, <0.5; additivity, 0.5-2; antagonism,>2.

^b^n, no. of fresh field isolates tested.

^c^n, no. of repeats.

Geometric mean IC_50_ and IC_90_ in nM for mirincamycin (MIR), tafenoquine (TQ), chloroquine (CHL) and dihydroartemisinin (DHA) as well as the sum of fractional inhibitory (ΣFIC) concentrations for *P. falciparum* field isolates and culture clones 3D7 (chloroquine-sensitive) and K1 (chloroquine-resistant) tested in checkerboards assays.

Out of a total of 55 patient samples 43 (78.2%; 95% confidence interval [CI]: 64.6 to 87.8) were successfully tested with a geometric mean parasite density of 7,646 per μL (95% CI: 5,399 to 10,827). The geometric mean of the 50% inhibitory concentration (IC_50_) for *trans*-mirincamycin was 1,212.6 nM (N = 43; 95% CI: 703.9 to 2,088.9) and the corresponding value for clindamycin was 880.4 nM (N = 43; 95% CI: 469.7 to 1,650.1). IC_50_, IC_90_, IC_99_ values with 95% confidence intervals for all drugs tested are shown in Table 2. No correlation was found between parasite density and inhibitory concentrations (IC_50_) of mirincamycin (R = 0.14; P = 0.4) and clindamycin (R = 0.10; P = 0.5) suggesting little influence of the inoculum size on the validity of *in vitro* assays. Individual IC values were calculated for all drugs tested in parallel and compared by nonparametric correlation analysis to determine potential cross-sensitivity and/or cross-resistance patterns between the drugs. Mirincamycin showed significant activity correlation with clindamycin (R = 0.64; P = 0.0002; N = 28) and azithromycin (R = 0.390; P = 0.040; N = 28) but no evidence of a correlation with any of the other tested anti-malarials (dihydroartemisinin: R = 0.269; P = 0.166; N = 28, mefloquine: R = 0.031; P = 0.875; N = 28, quinine: R = 0.025; P = 0.901; N = 28 and chloroquine: R = 0.319; P = 0.098; N = 28).

**Table 2:** **Geometric mean inhibitory concentration of various drugs against fresh *P. falciparum *** **isolates from Bangladesh**

**Antiplasmodial activity [nm] (95% CI)**			
**Drug (n**^**a**^**)**^**b**^	**IC**_**50 **_**(95% CI)**	**IC**_**90 **_**(95% CI)**	**IC**_**99 **_**(95% CI)**
MIR (n = 43)	1,212.6 (703.9 – 2088.9)	12,116.4 (6296.4 – 23 316.0)	34,329.2 (18 895.1 – 62 370.2)
CLI (n = 43)	880.4 (469.7 – 1650.1)	12,074.7 (6470.9 – 22 531.2)	36,581.3 (24 386.7 – 54 873.9)
AZM (n = 43)	4,082.6 (2947.5 – 5654.8)	21,781.4 (17 004.1 – 27 901.0)	39,979.5 (26 901.2 – 59 415.9)
DHA (n = 42)	0.9 (0.7 – 1.1)	2.9 (2.2 – 4.0)	4.8 (3.3 – 6.9)
CHL (n = 42)	102.6 (78.0 –134.9)	370.6 (279.1 – 492.1)	651.2 (467.1 – 907.9)
QNN (n = 42)	61.2 (46.6 – 80.2)	289.4 (236.2 – 354.7)	538.5 (421.2 – 688.5)
MEF (n = 42)	13.6 (9.3 – 20.0)	64.9 (47.1 – 89.3)	272.7 (38.1 – 1953.9)

^a^n, no. of fresh field isolates tested.

^b^MIR, mirincamycin; CLI, clindamycin; AZM, azithromycin; DHA, dihydroartemisinin; CHL, chloroquine; QNN, quinine; MEF, mefloquine.

IC_50_s for trans-mirincamycin and clindamycin were 111 and 79 times higher after only 24 h incubation than after 72 h incubation, suggesting a slow mode of action in malaria parasites.

## Discussion

So far there is only limited evidence for the *in vitro*[6] and *ex vivo* activity [9] of mirincamycin. *P. cynomolgi* animal models have proven that mirincamycin was curative even when given as monotherapy [3,4]. Interestingly, the substance seems to show considerable activity against hypnozoites [4], and may enhance the effect of primaquine when given in combination [5].

For the first time, these results prove that mirincamycin has an additive mode of interaction *in vitro* with a slight trend to synergism when combined with various standard anti-malarials. Similar drug interaction profiles were found 2003 by Ramharter *et al.* for clindamycin in combination with DHA where effective concentrations of clindamycin were comparable to our results for mirincamycin [15]. Subsequently conducted clinical trials have proven high efficacy of clindamycin in combination with conventional anti-malarial drugs [16]. Clinical studies will need to show in how far this is also the case for mirincamycin.

8-aminoquinolines (like primaquine and tafenoquine) can cause severe haemolysis in individuals with G6PD deficiency. The challenge is therefore either to improve the efficacy and thereby potentially reduce the dose of 8-aminoquinolines with new combination partners possibly resulting in a reduced risk of haemolysis or to overcome this side effect with safer replacement drugs.

Mirincamycin alone or in combination (e.g. with 8-aminoquinolines) may be a promising candidate for malaria prophylaxis in nonimmune subjects, such as tourists and soldiers and could potentially help to enhance the hypnozoitocidal activity of 8-aminoquinolines by concomitant administrion, as previously demonstarted in a *P. cynomolgi* model [4,5]. A lower dose could potentially also result in improved safety of 8-aminoquinolines (e.g. in glucose-6-phosphatedehydrogenase-deficient subjects).

Limitations of this study include all potential shortcomings of an *in vitro* study as well as the potential bias arising from the fact that only *P. falciparum* has been tested which cannot be extrapolated to *P. vivax* or *P. ovale* in the absence of a well-established culture model [17]. Also among 8-aminoquinolines only tafenoquine was tested in this study, which in recent studies has demonstrated high efficacy in the treatment and relapse prevention of vivax malaria in combination with chloroquine [18]. However, currently primaquine remains the only 8-aminoquinolone widely used for *P. vivax* radical cure.

## Conclusions

The study showed that mirincamycin has additive to synergistic interaction in combination with different classes of anti-malarials, exhibits no activity correlation with traditional anti-malarials and exerts substantial anti-malarial activity on its own. As a consequence mirincamycin may be a potential candidate for clinical exploration either in combination with faster acting anti-malarials in the treatment of multidrug-resistant falciparum malaria or in combination with other drugs for the treatment of non-falciparum malaria.

## Competing interests

The authors have declared that they have no competing interests.

## Authors’ contributions

HN contributed to all steps from elaboration to the final review (study design, study coordination, overall supervision, data analysis and manuscript review). H-PF, DG PS and PSw carried out the *in vitro* studies. PS and PSw supervised and carried out patient samples collection in the field. PS wrote the first draft of the manuscript. PSw and H-PF contributed to the writing of the manuscript. RH helped to design the study protocol, monitored laboratory quality and corrected the manuscript. WAK participated in the coordination of patient samples collection in the field, drafted the manuscript and helped to analyze the data. WG helped to design the study protocol and revised the final manuscript. All authors read and gave their consent to the final manuscript.
